# Predicting perioperative myocardial injury/infarction after noncardiac surgery in patients under surgical and medical co-management: a prospective cohort study

**DOI:** 10.1186/s12877-024-05130-x

**Published:** 2024-06-21

**Authors:** Shaozhi Xi, Bin Wang, Yanhui Su, Yan Lu, Linggen Gao

**Affiliations:** Department of Comprehensive Surgery, The Second Medical Center & National Clinical Research Center for Geriatric Diseases, Chinese PLA General Hospital, No.28 Fu Xing Road, Beijing, 100853 China

**Keywords:** Perioperative myocardial injury/Infarction, Noncardiac surgery, Surgical and medical co-management, Risk prediction

## Abstract

**Background:**

Perioperative myocardial injury/infarction (PMI) following noncardiac surgery is a frequent cardiac complication. This study aims to evaluate PMI risk and explore preoperative assessment tools of PMI in patients at increased cardiovascular (CV) risk who underwent noncardiac surgery under the surgical and medical co-management (SMC) model.

**Methods:**

A prospective cohort study that included consecutive patients at increased CV risk who underwent intermediate- or high-risk noncardiac surgery at the Second Medical Center, Chinese PLA General Hospital, between January 2017 and December 2022. All patients were treated with perioperative management by the SMC team. The SMC model was initiated when surgical intervention was indicated and throughout the entire perioperative period. The incidence, risk factors, and impact of PMI on 30-day mortality were analyzed. The ability of the Revised Cardiac Risk Index (RCRI), frailty, and their combination to predict PMI was evaluated.

**Results:**

613 eligible patients (mean [standard deviation, SD] age 73.3[10.9] years, 94.6% male) were recruited consecutively. Under SMC, PMI occurred in 24/613 patients (3.9%). Patients with PMI had a higher rate of 30-day mortality than patients without PMI (29.2% vs. 0.7%, *p* = 0.00). The FRAIL Scale for frailty was independently associated with an increased risk for PMI (odds ratio = 5.91; 95% confidence interval [CI], 2.34–14.93; *p* = 0.00). The RCRI demonstrated adequate discriminatory capacity for predicting PMI (area under the curve [AUC], 0.78; 95% CI, 0.67–0.88). Combining frailty with the RCRI further increased the accuracy of predicting PMI (AUC, 0.87; 95% CI, 0.81–0.93).

**Conclusions:**

The incidence of PMI was relatively low in high CV risk patients undergoing intermediate- or high-risk noncardiac surgery under SMC. The RCRI adequately predicted PMI. Combining frailty with the RCRI further increased the accuracy of PMI predictions, achieving excellent discriminatory capacity. These findings may aid personalized evaluation and management of high-risk patients who undergo intermediate- or high-risk noncardiac surgery.

## Introduction

Perioperative myocardial injury/infarction (PMI) following noncardiac surgery has been increasingly recognized as a frequent cardiac complication, mostly without typical ischemic symptoms [1–3], which occurs in at least 8% of elective procedures [2, 4], and 20% of major surgeries [5]. Moreover, PMI is independently associated with an increased risk of cardiovascular (CV) morbidity and mortality at 30 days and up to 1 year after noncardiac surgery [1, 6–9]. Asymptomatic PMI is comparable to symptomatic PMI [1]. Therefore, strategies to improve the detection of PMI for noncardiac surgical patients may provide major medical benefits [10]. For early identification of surgical patients at high risk of PMI, attention should be paid to preoperative assessment. The Revised Cardiac Risk Index (RCRI) is recommended in clinical practice guidelines [10] and has been widely used to predict perioperative cardiac risk [11–14]. Meanwhile, frailty is an emerging risk factor for major adverse CV events perioperatively among patients undergoing noncardiac surgery [15]. However, it remained unknown whether the RCRI and frailty could perform well for the prediction of PMI in noncardiac surgery patients under care of the surgical and medical co-management (SMC) model.

The complexity of patients undergoing intermediate- or high-risk noncardiac surgery exceeds the capacity of any one surgical group to be managed independently, so patients benefit from a team-based approach to care [16]. The collaborative model of SMC [17] is a strategy that emphasizes patient-centered care and aims to optimize the quality of perioperative care and improve survival and postoperative outcomes. Under SMC, internists are proactive involved in all aspects of perioperative care, including preoperative assessment and optimizing perioperative medical therapy, postoperative support and management of complications, and maximizing functional recovery [18]. Previous studies have reported that the implement of SMC resulted in better outcomes in high-risk orthopedic [19–21], vascular [22], colorectal surgery [23] and neurosurgery [24, 25], such as reduced medical complications, decreased length-of-stay, reduced in-hospital mortality or one-year mortality [26–28]. The practice of SMC is gaining popularity, as there has been a rapid increase in the percentage of noncardiac surgical patients under SMC, mainly in the United States [29]. However, little is known about the risk of PMI in intermediate- or high-risk noncardiac surgery patients under care of the SMC model in a real-world clinical setting.

The Department of Geriatric Comprehensive Surgery of the author’s team is a perioperative SMC team with 35 years of clinical experience in perioperative management. Thus, in this study, we evaluated PMI risk and explore preoperative assessment tools of PMI in patients at increased CV risk who underwent intermediate- or high-risk noncardiac surgery under care of the SMC model in a real-world clinical setting.

## Methods

### Study population

This was a prospective cohort study that included consecutive patients at increased CV risk who underwent intermediate- or high-risk inpatient noncardiac surgery at the Second Medical Center, Chinese PLA General Hospital, Beijing, China, between January 2017 and December 2022. Patients were included if they were ≥ 65 years old, or ≥ 50 years old with a history of cardiovascular disease (CVD) or CV risk factors (e.g., smoking, obesity, hypertension, diabetes, and dyslipidemia) [30], treated with intermediate- or high-risk noncardiac surgery according to the criteria of the ESC/ESA surgical risk score [10] and with a postoperative stay of ≥ 2 days. Plasma concentrations of cardiac troponin (cTn) were measured routinely within 14 days before surgery, on postoperative days 1 and 2, and later if clinically indicated. Twelve-lead electrocardiogram (ECG) was performed when PMI was detected and whenever indicated clinically. A detailed clinical evaluation for the PMI work-up and therapy was performed when PMI was detected.

Patients were excluded if one of the following was met: cardiac surgery or MI within 14 days before admission, surgery involving a cardiac surgeon (e.g., coronary artery bypass graft, heart valve surgery, repair of congenital heart disease, surgery with cardiopulmonary bypass, or cardiac transplantation), low-risk noncardiac surgery, no cTn measurement within 14 days before surgery or 3 days after surgery, elevated preoperative cTn level, or lost to follow-up after discharge.

### Surgical and medical co-management

In the Second Medical Center of Chinese PLA General Hospital, the author’s team is a perioperative SMC team with 35 years of clinical experience in perioperative management. The relationship between the surgeon and the internist is a patient-centered collaboration. Under normal conditions, the internist also participates in daily rounds, prescribes medicines, and writes progress notes.

**Fig. 1:**
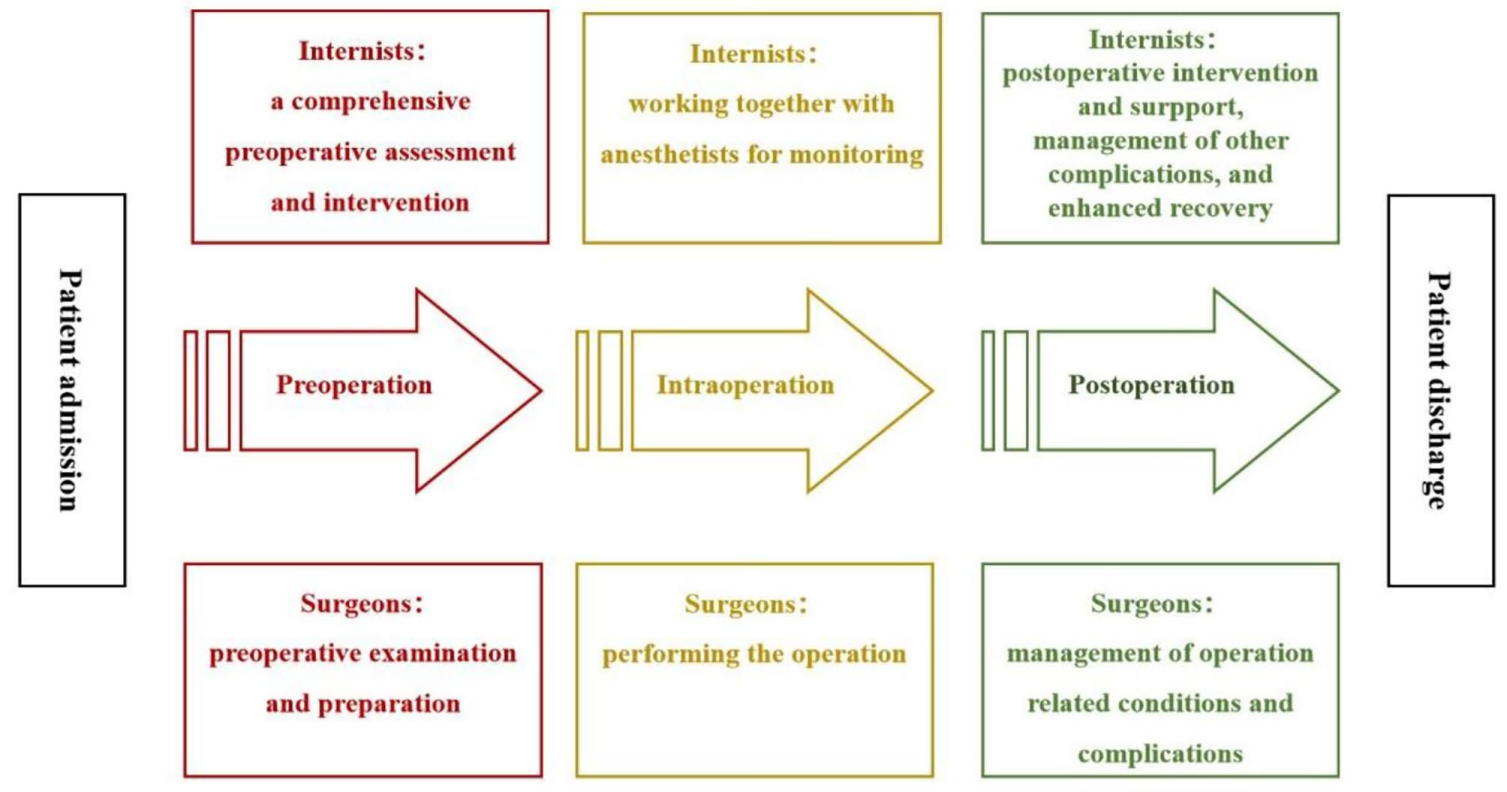
Flow diagram of surgical and medical co-management (SMC)

All patients were treated by the perioperative SMC team according to the updated clinical guidelines, among which the guidelines for CV assessment and management of patients were inspired by the 2022 ESC guidelines [10]. It is recommended to routinely measure cTn in patients with an increased CV risk before intermediate- and high-risk noncardiac surgery, and 24 and 48 h thereafter. It is also recommended to obtain a preoperative 12-lead ECG, and measure preoperative brain natriuretic peptide (BNP) concentrations in those patients. As Fig. 1 shows, SMC was initiated when surgical intervention was indicated and throughout the entire perioperative period.

### Ethics statement

This study was approved by the Clinical Research Ethics Committee of Chinese PLA General Hospital and registered with the ISRCTN registry (reference number: ISRCTN58897355). The study was performed following the Declaration of Helsinki. Written informed consent was obtained from all included patients.

### Variables and definition

The data of the included patients was prospectively collected and recorded. Variables included baseline demographics, concomitant diseases, the American Society of Anesthesiologists (ASA) score, perioperative laboratory values such as hemoglobin, creatinine, and cTn, examination indices such as ECG and cardiac ultrasound, type of surgical procedure, surgical information, perioperative medications, and postoperative complications during hospitalization. Thirty-day mortality was determined by phone interview or clinical visit.

We calculated the RCRI for each patient to evaluate the risk of cardiac complications. The RCRI [11] consists of six categorical variables, including a history of ischemic heart disease, history of heart failure, history of insulin-dependent diabetes mellitus, history of cerebrovascular accident or transient ischemic attack (TIA), baseline creatinine ≥ 2 mg/dl, and high-risk surgery (intrathoracic, intra-abdominal, or suprainguinal vascular surgery). One point was given for each variable, and the RCRI score was calculated for each patient by summing all of the points. Patients were divided into four risk classes (I: 0 point; II: 1 point; III: 2 points; IV: ≥ 3 points).

Frailty was assessed using the FRAIL Scale [31, 32], which consists of a 5-item screening questionnaire with simple “yes” or “no” answers: Fatigue (evaluated by asking patients if they felt tired most of the time), muscle resistance (indicated by a patient’s capacity to climb a flight of stairs), aerobic capacity (evaluated by a patient’s capacity to walk one block independently), illness (evaluated by the presence of five or more diseases), and weight loss (measured by a decline of ≥ 5% within the past 6 months). One point was given for each affirmative response. Scores ranged from 0 to 5 points, and patients were divided into three categories: robust (0 point), prefrail (1–2 points), and frail (≥ 3 points).

### Outcomes

The primary outcome of PMI was a peak plasma concentration of cTn T ≥ 30 ng/L (measured by using an Elecsys System, Roche Diagnostics) within 3 days following noncardiac surgery, independent of symptoms or ECG changes [2, 33]. According to the dominant trigger for MI injury, PMI was classified as extra-cardiac (triggered by a primarily extra-cardiac disease, such as severe sepsis, stroke, pulmonary embolism [PE], or surgical trauma) or cardiac (type 1 myocardial infarction [TIMI] caused by plaque rupture, type 2 myocardial infarction [T2MI] caused by a supply-demand mismatch, acute heart failure, or tachyarrhythmia) [7]. The confirmation and classification of PMI were adjudicated by two independent internists based on all related clinical information.

Other postoperative complications (e.g., the Clavien-Dindo classification of surgical complications [34, 35]) and thirty-day mortality were also collected.

### Statistical analysis

Continuous variables are presented as mean with standard deviation (SD) and were compared using Student’s *t*-test. Categorical variables are reported as numbers with percentages and were compared using the chi-square test or Fisher’s exact test for categorical variables, as appropriate.

Logistic regression analysis was used for univariate and multivariate analyses, with PMI as the dependent variable. Potential predictors of PMI were selected using univariate analysis (*p* < 0.1). The potential predictors and other clinically related candidate variables were included in the multivariate analysis, and independent PMI predictors were selected using multivariate forward selection (*p* < 0.05).

A receiver operating characteristic (ROC) curve was used to evaluate the discriminatory ability of the RCRI, frailty, and their combination to predict PMI. An area under the curve (AUC) > 0.7 was considered to indicate acceptable discriminatory capacity, with AUC ≥ 0.8 considered to indicate excellent discriminatory capacity [11, 36]. Calibration was assessed using the Hosmer-Lemeshow goodness-of-fit test (HL), and *p* < 0.05 indicated a poor fit.

In addition, post-hoc sensitivity analyses were performed. In the first sensitivity analysis, we assessed the performance of the RCRI, frailty, and their combination for predicting PMI in the subgroups of elderly patients, patients with specific comorbidities, patients with high-risk surgery, and those with ASA status ≥ 3. In the second sensitivity analysis, we assessed the performance of the RCRI, frailty, and their combination for predicting PMI in whom with high-risk surgery and those with intermediate-risk surgery. There were no patients with low-risk surgery. In the third sensitivity analysis, we assessed the performance of the RCRI, frailty, and their combination for predicting PMI in whom aged ≥ 75 years and those aged < 75years.

All calculations were performed with SPSS software (version 25.0; IBM Corp., Armonk, NY, USA).

## Results

### Study cohort and baseline characteristics

**Fig. 2:**
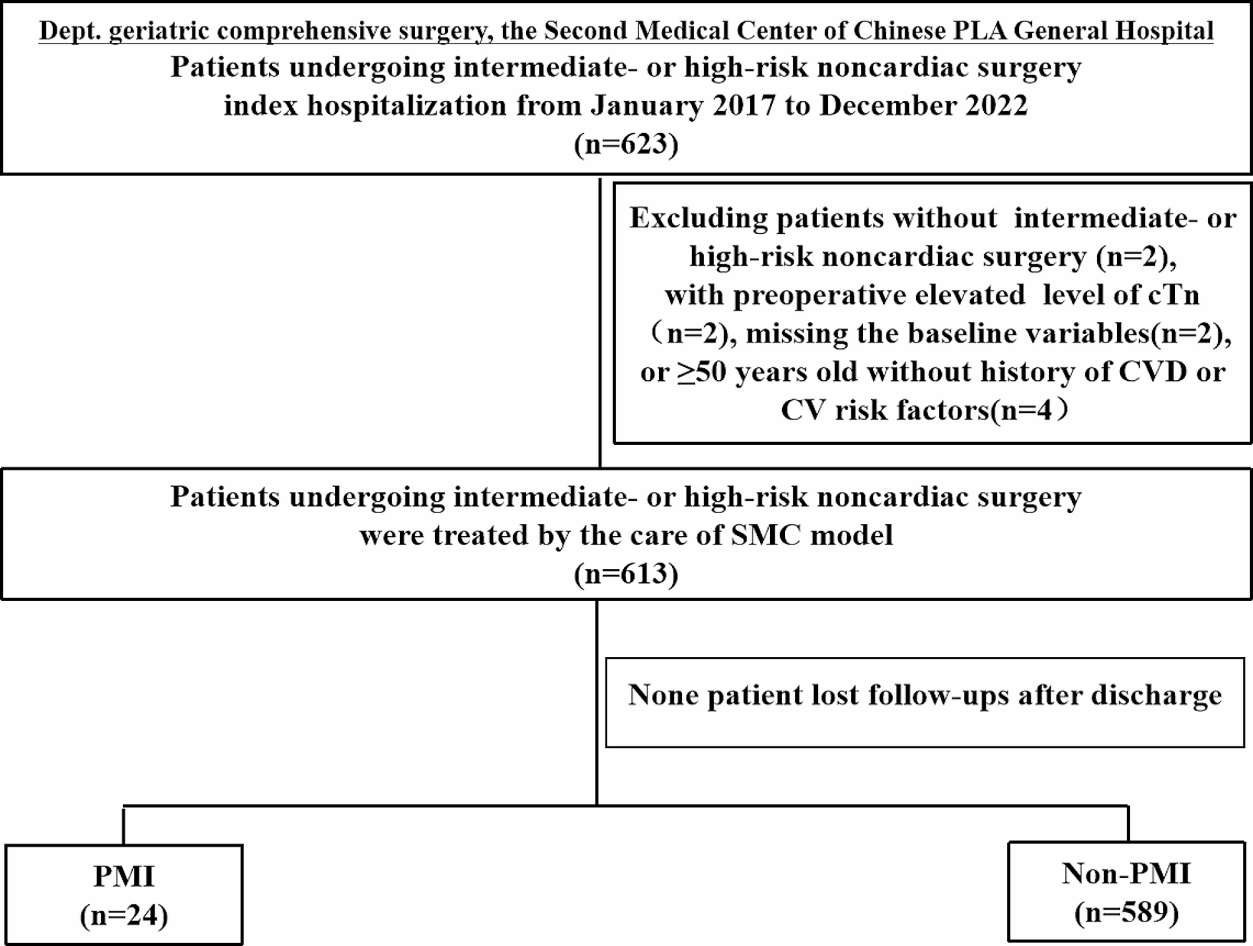
Study flow chart. CVD, cardiovascular disease; CV, cardiovascular; SMC, surgical and medical co-management; PMI, perioperative myocardial injury/infarction

A total of 613 eligible patients were included in this analysis (Fig. 2). In total, the mean (SD) age was 73.3 (10.9) years, with 466 patients aged ≥ 65 years (76.0%). Meanwhile, 183 patients had coronary artery disease (CAD) (29.8%), and 51 had undergone a coronary intervention or coronary artery bypass grafting (CABG) (8.4%), of whom 3 patients underwent coronary revascularization (with 1 patient underwent percutaneous transluminal coronary angioplasty and 2 underwent CABG) before the scheduled surgical procedure as part of the reduction of cardiac risk (0.5%). Moreover, 594 patients (97.0%) received an ECG, and the biomarker BNP was measured before noncardiac surgery in 561 patients (91.5%). In brief, 24 patients (3.9%) had a Clavien-Dindo classification for surgical complications ≥ 3. The overall 30-day mortality rate was 1.7% (*n* = 11/613).

### Incidence and the underlying PMI etiologies

PMI occurred in 24/613 patients (3.9%). The underlying PMI etiologies were extra-cardiac in 6/24 (25.0%) patients, TIMI in 5/24 (20.8%) patients, acute heart failure in 4/24 (16.6%) patients, tachyarrhythmia in 5/24 (20.8%), and likely T2MI in 4/24 (16.6%).

### Risk factors for PMI

**Fig. 3:**
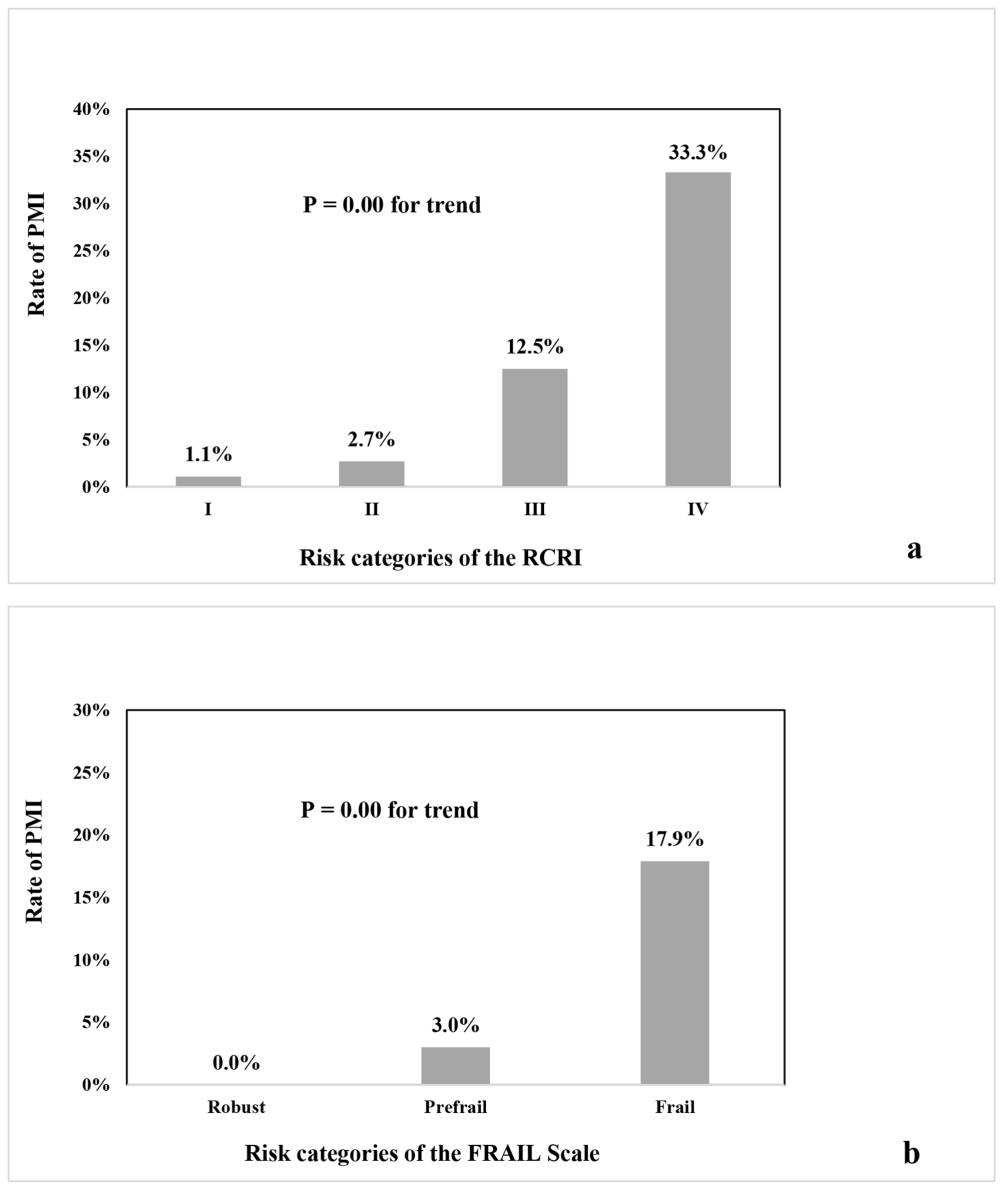
Ratings of PMI across risk categories of the RCRI (**a**) and FRAIL scale (**b**). *P*-value is for the comparison across the risk categories of the scoring systems

Patients with PMI were older, had a lower body mass index (BMI) and hemoglobin level, had more CV comorbidities, and had a higher likelihood of ASA status ≥ 3 and emergency noncardiac surgery (Table 1). Moreover, the rate of PMI increased with the risk category of the RCRI and FRAIL scale systems (Fig. 3). Consequently, patients with PMI had a much higher rate of 30-day mortality than patients without PMI (29.2% vs. 0.7%, *p* = 0.00).

**Table 1:** Characteristics of the study population

	PMI (*n* = 24)	Non-PMI (*n* = 589)	*p*-value
Age, (mean ± SD), y	82.2 ± 8.3	72.9 ± 10.9	0.00
Male, n (%)	22 (91.7%)	558 (94.7%)	0.85
Han race, n (%)	23 (95.8%)	574 (97.5%)	1.00
BMI, (mean ± SD), kg/m^2^	23.0 ± 3.2	24.8 ± 2.6	0.00
Current smoker, n (%)	4 (16.7%)	98 (16.6%)	1.00
Current drinker, n (%)	3 (12.5%)	71 (12.1%)	1.00
Medical history, n (%)			
Cardiac diseases	18 (75.0%)	211 (35.8%)	0.00
Coronary artery disease	18 (75.0%)	165 (28.0%)	0.00
Hypertension	20 (83.3%)	376 (63.8%)	0.05
Chronic heart failure	2 (8.3%)	3 (0.5%)	0.01
Diabetes mellitus	9 (37.5%)	185 (31.4%)	0.53
Diabetes mellitus, insulin-dependent	3 (12.5%)	39 (6.6%)	0.48
History of stroke/TIA	5 (20.8%)	47 (8.0%)	0.07
COPD	3 (12.5%)	24 (4.1%)	0.14
Chronic kidney disease	6 (25.0%)	34 (5.8%)	0.00
ASA status ≥ 3, n (%)	24 (100%)	361 (61.3%)	0.00
FRAIL score, n (%)			0.00
0	0 (0.0%)	210 (35.7%)	
1	9 (37.5%)	302 (51.3%)	
2	1 (4.2%)	13 (2.2%)	
3	1 (4.2%)	10 (1.7%)	
4	12 (50.0%)	53 (9.0%)	
5	1 (4.2%)	1 (0.2%)	
Frailty (FRAIL score ≥ 3)	14 (58.3%)	64 (10.9%)	0.00
RCRI class, n (%)			0.00
I	3 (12.5%)	263 (44.7%)	
II	7 (29.2%)	253 (43.0%)	
III	9 (37.5%)	63 (10.7%)	
IV	5 (20.8%)	10 (1.6%)	
Emergency surgery, n (%)	7 (29.2%)	25 (4.2%)	0.00
Admission data and laboratory evaluation			
Heart rate (mean ± SD), bpm	75.8 ± 16.3	71.0 ± 11.4	0.20
LVEF (mean ± SD), %	57.8 ± 9.4	62.8 ± 3.8	0.03
Hemoglobin, (mean ± SD) g/L	117.1 ± 25.2	134.6 ± 14.7	0.00
< 110 g/L	6 (25.0%)	30 (5.1%)	0.00
WBC (mean ± SD) × 10^9^/L	7.3 ± 2.3	6.7 ± 3.1	0.31
AST (mean ± SD), U/L	25.2 ± 22.4	21.5 ± 17.4	0.31
Serum creatinine (mean ± SD), mg/dl	104.4 ± 62.2	80.2 ± 28.3	0.08
≥ 2 mg/dL	4 (16.7%)	8 (1.4%)	0.00
Preoperative medication, n (%)			
Antithrombotic therapy			0.14
Antiplatelet	12 (50.0%)	179 (30.4%)	
Anticoagulant	1 (4.2%)	25 (4.2%)	
None	11 (45.8%)	385 (65.4%)	
Bridging anticoagulation after admission	12 (50.0%)	159 (27.0%)	0.01
Statins	18 (75.0%)	360 (61.1%)	0.17
Β-blockers	10 (41.7%)	171 (29.1%)	0.19
Calcium channel blockers	10 (41.7%)	242 (41.1%)	0.96
ACEI or ARBs	7 (29.2%)	202 (34.3%)	0.60
Blood transfusion	1 (4.2%)	22 (3.7%)	1.00
30-day mortality, n (%)	7 (29.2%)	4 (0.7%)	0.00

**Abbreviations:** PMI, perioperative myocardial injury/infarction; SD, standard deviation; BMI, body mass index; TIA, transient ischemic attack; COPD, chronic obstructive pulmonary disease; ASA, American Society of Anesthesiologists; RCRI, the Revised Cardiac Risk Index; LVEF, left ventricular ejection fraction; WBC, white blood cells; AST, aspartate aminotransferase; ACEI, angiotensin-converting enzyme inhibitor; ARBs, angiotensin receptor blockers

Multivariate regression analysis revealed that frailty (odds ratio [OR] = 5.91; 95% CI, 2.34–14.93; *p* = 0.00), coronary heart disease (OR = 4.69; 95% CI, 1.75–12.58; *p* = 0.00), and hemoglobin (OR = 0.97; 95% CI, 0.94–0.99; *p* = 0.01 were independently associated with PMI (Table 2).

**Table 2:** Multivariate predictors of PMI after noncardiac surgery

Variables	Adjusted OR (95% CI)	*p*-value
Frailty	5.91 (2.34–14.93)	0.00
Coronary artery disease	4.69 (1.75–12.58)	0.00
Hemoglobin	0.97 (0.94–0.99)	0.01

**Abbreviations:** PMI, perioperative myocardial injury/infarction; OR, odds ratio; CI, confidence interval

### Ability of the RCRI, frailty, and their combination to predict PMI

The calibrated RCRI score, frailty, and their combination were adequate to predict PMI in the whole cohort (HL *p* > 0.05). The RCRI demonstrated acceptable discriminatory capacity for predicting PMI (AUC, 0.78; 95% CI, 0.67–0.88). Frailty also demonstrated acceptable discriminatory capacity (AUC, 0.74; 95% CI, 0.62–0.86). Combining frailty with the RCRI further increased the AUC to 0.87 (95% CI, 0.81–0.93) for predicting PMI among all patients (Table 3).

**Table 3:** Assessment of the RCRI, frailty, and the frailty plus RCRI combination as predictors of PMI in the whole study population and in subgroups

Groups	PMI, *n* (%)		RCRI	Frailty	Frailty plus RCRI
The whole cohort		3.9% (24/613)	0.78 (0.67–0.88)	0.74 (0.62–0.86)	0.87 (0.81–0.93)
Cardiac diseases		7.9% (18/229)	0.76 (0.64–0.88)	0.70 (0.56–0.84)	0.82 (0.72–0.92)
Coronary artery disease		9.8% (18/183)	0.71 (0.58–0.85)	0.69 (0.55–0.83)	0.78 (0.67–0.90)
Coronary intervention/CABG		19.6% (10/51)	0.74 (0.55–0.93)	0.70 (0.50–0.91)	0.78 (0.60–0.96)
Hypertension		5.1% (20/396)	0.81 (0.72–0.91)	0.71 (0.58–0.85)	0.86 (0.78–0.93)
Diabetes mellitus		4.6% (9/194)	0.81 (0.68–0.94)	0.74 (0.53–0.94)	0.86 (0.73–0.99)
ASA status ≥ 3		6.2% (24/385)	0.73 (0.62–0.84)	0.71 (0.59–0.83)	0.81 (0.74–0.89)
High-risk surgery		6.2% (10/161)	0.81 (0.69–0.94)	0.73 (0.53–0.93)	0.86 (0.73–0.99)
Intermediate-risk surgery		3.1% (14/452)	0.75 (0.60–0.89)	0.75 (0.60–0.90)	0.87 (0.81–0.93)
Age ≥ 75 y		7.8% (21/270)	0.74 (0.61–0.86)	0.67 (0.54–0.80)	0.80 (0.71–0.89)
Age < 75y		0.9% (3/343)	0.82 (0.64–0.99)	0.82 (0.50-1.00)	0.90 (0.73-1.00)

**Abbreviations:** PMI, perioperative myocardial injury/infarction; RCRI, the Revised Cardiac Risk Index; CABG, coronary artery bypass grafting; ASA, American Society of Anesthesiologists

### Post-hoc sensitivity analyses

The calibrated RCRI score, frailty, and their combination were adequate to predict PMI in all subgroups (HL *p* > 0.05). The RCRI demonstrated acceptable discriminatory capacity for elderly patients, patients with cardiac diseases, CAD patients, coronary intervention/CABG patients, and those with ASA status ≥ 3 (AUC > 0.7) (Table 3). Meanwhile, the RCRI had excellent discriminatory power for subgroups of patients with hypertension, diabetes mellitus, and high-risk surgery (AUC ≥ 0.8) (Table 3). In addition, frailty showed poor or acceptable discriminatory power for all subgroups (AUC, 0.67–0.74) (Table 3). Their combination enhanced the discriminatory power for all subgroups (AUC, 0.78–0.86) versus the RCRI alone (Table 3).

The discrimination by the RCRI was acceptable or excellent in both settings (patients with high-risk surgery and those with intermediate-risk surgery) (AUC > 0.7) (Table 3). Discrimination performance of frailty remained consistent in both settings (AUC > 0.7) (Table 3). Their combination also enhanced the discriminatory power for the both settings (AUC > 0.8) (Table 3).

Whether patients aged ≥ 75 years or not, the discrimination performance of the RCRI, frailty, and their combination for predicting PMI also largely remained consistent with those of the whole cohort (Table 3).

## Discussion

The main findings of this study are that, under SMC, the incidence of PMI (3.9%) was lower than that observed in the VISION study [3, 37–40], among others [1, 2, 7], in which the incidence ranged from 8 to 19%. Patients with PMI had a much higher 30-day mortality rate than those without PMI. Frailty was independently associated with an increased risk for PMI. The RCRI demonstrated adequate discriminatory capacity for predicting PMI. The combination of frailty and the RCRI further increased the accuracy for predicting PMI, with excellent discriminatory capacity. To the best of our knowledge, this is the first study to evaluate PMI risk in SMC-treated high-risk patients undergoing intermediate- or high-risk noncardiac surgery. The findings may facilitate personalized evaluations and management of high-risk patients undergoing intermediate- or high-risk noncardiac surgery.

Attention should be paid to preoperative assessment to identify surgical patients at high risk of CV complications, to minimize the incidence and impact of PMI. Besides an accurate history and a clinical examination, preoperative assessment tools are also recommended [10–13]. The RCRI was developed to estimate the risk of 30-day mortality, MI, or cardiac arrest, and is easy to use [14]. The RCRI has been widely used to predict perioperative cardiac complications [10–14]. In external validation studies, the RCRI showed moderate discriminatory capacity and high negative predictive value in noncardiac surgery patients [41–43]. However, it underestimates the risk of major adverse cardiac events [42–44]. In our study, under SMC, the RCRI had acceptable discriminatory capacity for predicting PMI in patients with increased CV risk undergoing intermediate- and high-risk noncardiac surgery.

As we know, there are gender-dependent clinical phenotypes of comorbidities and risk factors. Gender may significantly affect the assessment and management of patients with specific disease undergoing noncardiac surgery [10]. Whereas, there is lack of well- powered studies to evaluate the interplay between gender, age and comorbidities in patients undergoing noncardiac surgery. Evidence regarding the gender-specific assessment and management strategies for patients undergoing noncardiac surgery is still lacking. The VISION study showed that the event incidence and discrimination performance of RCRI was similar for men and women [11]. Consistent with the VISION study [11], our study found that PMI occurred in 22/580 male patients (3.8%), and 2/33 female patients (6.1%). There was no significant difference in gender between patients with PMI and patients without PMI (*p* = 0.85). The RCRI had an AUC of 0.76(95% CI, 0.65–0.87) for PMI in men and 0.99(95% CI, 0.96-1.00) in women. Whereas, most of the patients were male in our study, additional studies are needed to investigate the gender differences in the assessment and management of patients undergoing noncardiac surgery.

Frailty, an aging-related decrease in functional status and physiological reserves is associated with multiple morbidities. It is an emerging risk factor for adverse outcomes in older surgical patients [10, 45]. Recent studies showed that frailty may contribute to and result from CVD [46, 47]. It has also been shown to be associated with an increased risk for major adverse CV events perioperatively among adults undergoing noncardiac surgery in the United States, by a study based on a large nationwide database [15]. Consistent with previous studies [10, 15, 45], the FRAIL scale for frailty was independently associated with PMI in the present SMC-treated high-risk adult patients who underwent noncardiac surgery. In total, 76.0% of our sample were elderly (aged ≥ 65 years), and the dominant trigger for PMI was extra-cardiac or T2MI, which may explain the association between frailty and PMI. In addition, the discriminatory power for PMI was improved by combining frailty with the RCRI. Thus, the findings indicate that adding frailty to the RCRI may provide a more accurate guide to physicians concerning the required therapy. Patients will receive benefits and avoid being exposed to unnecessary risk.

The guideline-recommended CVD treatment and CV risk factors should be optimized before noncardiac surgery [10]. The SMC model aids risk-reduction strategies, improves clinical outcomes, and reduced postoperative CV complications in our study. General guideline-recommended risk-reduction strategies include: First, CV risk factor and lifestyle interventions, including controlling blood pressure, dyslipidemia, and diabetes, and stopping smoking > 4 weeks before surgery. Second, optimizing perioperative medical therapy, including beta-blockers, statins, calcium channel blockers, angiotensin-converting-enzyme inhibitors (ACEIs) or angiotensin receptor blockers (ARBs), and antithrombotic therapy. In our study, patients with PMI had more CV risk factors or CAD, with higher rates of preoperative antiplatelet therapy and bridging anticoagulation after admission than patients without PMI. Third, blood management. Hemoglobin should be measured, and anemia should be treated, preoperatively in patients scheduled for intermediate- and high-risk noncardiac surgery [10]. We routinely measured hemoglobin in our study and found that the baseline hemoglobin level was independently associated with PMI. The frequency of preoperative blood transfusion was not significantly different between patients with and without PMI. Fourth, perioperative management based on the specific type of CVD. Above all, we hope to continue to advance the standard of SMC and improve CV outcomes for high-risk patients undergoing noncardiac surgery by integrating new evidence with the updated guideline recommendations [48].

### Study limitations

Several limitations should be discussed. First, it was a single-center based prospective cohort study with a relatively small sample size (*N* = 613), however, this study focused on high-risk noncardiac surgical patients under SMC, which is representative and clinically relevant. In addition, the incidence rates of PMI across different types of surgery were similar to other studies [22, 49], which makes our findings credible (Table 4). Second, there is no unified definition of PMI. Our criterion was consistent with the PMI diagnostic criteria established based on a large international prospective cohort study of PMI [2]. Third, the underlying etiologies of PMI were inferred based on clinical criteria because most patients with PMI did not undergo coronary angiography. Fourth, this study included patients ≥ 65 years old, or ≥ 50 years old with a history of CVD or CV risk factors. As we know, specific clinical conditions can also increase the risk of CVD, such as anaemia, chronic kidney disease (CKD), cancer, chronic obstructive pulmonary disease (COPD), and mental disorders. Therefore, some patients with above mentioned specific clinical conditions might be overlooked. Whereas, in our study, the final cohort included 613 (98.4%) eligible patients after excluding patients without intermediate- or high-risk noncardiac surgery (*n* = 2), those with preoperative elevated level of cTn (*n* = 2) or missing the baseline variables(*n* = 2), and patients aged ≥ 50 years old without history of CVD or CV risk factors(*n* = 4) (Fig. 2). Among the four patients aged ≥ 50 years old without history of CVD or CV risk factors, none of them combined with anaemia, CKD, cancer, COPD, or mental disorders. Fifth, the inherent limitation of the study was based on its single-center and observational nature, as most of the patients were male and the management of patients is relatively homogenous.

**Table 4:** Postoperative outcomes of different types of noncardiac surgery

Type of surgery	PMI	30-day mortality
General surgery	6.6% (6/91)	4.4% (4/91)
Hepatobiliary	3.9% (5/127)	0.8% (1/127)
Urology	1.7% (2/119)	0.0% (0/119)
Orthopedic	0.8% (1/133)	1.5% (2/133)
Thoracic	1.2% (1/82)	0.0% (0/82)
Neurosurgery	27.3% (3/11)	9.1% (1/11)
Vascular surgery	17.1% (6/35)	8.6% (3/35)
Others	0.0% (0/15)	0.0% (0/15)

**Abbreviations:** PMI, perioperative myocardial injury/infarction

## Conclusions and implications

The incidence of PMI was relatively low in high CV risk patients undergoing intermediate- or high-risk noncardiac surgery under SMC. The RCRI performed adequately in terms of predicting PMI. Combining frailty and the RCRI further increased the accuracy of PMI predictions, achieving excellent discriminatory capacity.

## Data Availability

The raw data supporting the conclusions of this study will be available upon request from the corresponding authors.
